# Comparison of an automated DNA extraction and 16S rDNA real time PCR/sequencing diagnostic method using optimized reagents with culture during a 15-month study using specimens from sterile body sites

**DOI:** 10.1186/s12866-022-02542-w

**Published:** 2022-05-02

**Authors:** Konrad Egli, Martin Risch, Lorenz Risch, Thomas Bodmer

**Affiliations:** 1 Dr Risch, 3097 Liebefeld, Switzerland; 2 Dr Risch, Buchs, SG Switzerland; 3 Private University of the Principality of Liechtenstein, Triesen, Liechtenstein

**Keywords:** 16S rDNA real time PCR, DNA-free reagents, Evolution of methods, Mixed sequences, Additional benefit

## Abstract

**Background:**

16S rDNA-PCR for the identification of a bacterial species is an established method. However, the DNA extraction reagents as well as the PCR reagents may contain residual bacterial DNA, which consequently generates false-positive PCR results. Additionally, previously used methods are frequently time-consuming. Here, we describe the results obtained with a new technology that uses DNA-free reagents for automated DNA extraction and subsequent real time PCR using sterile clinical specimens.

**Results:**

In total, we compared 803 clinical specimens using real time PCR and culturing. The clinical specimens were mainly of orthopedic origin received at our diagnostic laboratory. In 595 (74.1%) samples, the results were concordant negative, and in 102 (12.7%) the results were concordant positive. A total of 170 (21.2%) clinical specimens were PCR-positive, of which 62 (36.5% from PCR positive, 7.7% in total) gave an additional benefit to the patient since only the PCR result was positive. Many of these 62 positive specimens were strongly positive based on crossingpoint values (54% < Cp 30), and these 62 positive clinical specimens were diagnosed as medically relevant as well. Thirty-eight (4.2%) clinical specimens were culture-positive (25 of them were only enrichment culture positive) but PCR-negative, mainly for *S. epidermidis*, *S. aureus* and *C. acnes*. The turnaround times for negative specimens were 4 hours (automated DNA extraction and real time PCR) and 1 working day for positive specimens (including Sanger sequencing). Melting-curve analysis of SYBR Green-PCR enables the differentiation of specific and unspecific PCR products. Using Ripseq, even mixed infections of 2 bacterial species could be resolved.

**Conclusions:**

For endocarditis cases, the added benefit of PCR is obvious. The crucial innovations of the technology enable timely reporting of explicit reliable results for adequate treatment of patients. Clinical specimens with truly PCR-positive but culture-negative results represent an additional benefit for patients. Very few results at the detection limit still have to be critically examined.

## Introduction

For the identification of bacterial species and/or genera, 16S rDNA PCR is a standard method in most clinical microbiology laboratories. For this analysis in particular, the available methods have changed during the past 30 years. The first reports in diagnostic microbiology starting at the end of the nineteen eighties describe the use of highly hazardous chemicals for the extraction of nucleic acids and detection of PCR products [1, 2]. For a long time, manual DNA extraction and non-real time amplification with subsequent electrophoretic separation of PCR products remained the state of the art [3–6]. Although real time PCR was developed many years ago, the application of 16S rDNA (including melting curve analysis for the differentiation of specific and nonspecific PCR products) came much later [7], probably because of the restriction of the maximal size of amplified fragments in real time PCR and because replacement of existing methods usually takes time. Manual DNA extraction is time-consuming and a potential source of contamination of processed clinical specimens. Conventional (not real time) PCR and subsequent electrophoresis are time-consuming as well, particularly since two rounds of long-lasting nested PCR are performed [3]. Therefore, the whole procedure of DNA extraction and PCR is prone to reagent contamination [4]. Reagent contamination also plays an important role when performing next-generation sequencing (NGS) of the 16S rRNA gene, since this obviously overestimates the number of sequences, especially for clinical specimens with a low number of species present [8]. An additional challenge is the excessive amount of human DNA, which influences DNA extraction and PCR [9]. To overcome these issues, researchers have lysed human cells, and DNA is degraded (with DNase) before lysis of bacterial species [10]. Even in usually sterile body sites, more than one microbial species can be present [11], or at the detection limit, slight contamination (which might also occur during sampling) can also cause mixed chromatograms. The online tool Ripseq [9] is very helpful for overcoming this issue. A limitation of Sanger sequencing is the fact that when bacterial species in the same clinical specimen demonstrate major differences in concentrations, only the most abundant species appear in the chromatogram [12, 13].

In this study, we therefore aimed to compare an improved automated technology (from Molzym) using DNA-free reagents (for DNA extraction and real time PCR) with conventional culture. The DNA extraction reagents used also remove human DNA during bacterial DNA extraction. This procedure (DNA-free reagents for automated DNA extraction as well as real time PCR and removal of human DNA) is a crucial new technology. With this new technology, negative results can be obtained within 4 hours (DNA extraction as well as real time PCR), and positive results can be obtained within 1 working day with minimal hands-on-time. This enables a timely reporting of high-quality results to the patient and is therefore a substantial improvement in comparison to previously applied methods. These two points (timely reporting and high-quality results) are essential for routine clinical diagnostics. Automated DNA extraction enables a faster diagnostics with a lower risk of contamination compared with manual DNA extraction [9] even with reagents of the same supplier. The vast majority of analyzed clinical specimens in this study were from orthopedic origin (e.g., implant-associated specimens, aspirates of joints, heart valves). The study was performed in the context of our accredited clinical routine diagnostics and describes the obtained results and available clinical information.

## Materials and methods

For DNA extraction, the Micro-Dx™ system (with CE IVD label for automated DNA extraction as well as PCR reagents) was used (Molzym, Germany) according to the package insert. This system allows fully automated DNA extraction and supplies reagents for subsequent real time PCR on LC480 (Roche, Switzerland). Both extraction reagents and PCR reagents were declared to be DNA free. The process includes DNase incubation for the removal of human DNA as well as DNA from already lysed bacterial cells at the beginning of DNA extraction. Generally, 1 mL of each liquid clinical specimen was used for extraction. Clinical specimens were from the clients of our officially certified medical routine laboratory (located in Switzerland) without exclusion of certain clinical specimens. The decision to choose 16S rDNA-PCR was made by clients (based on the recommendations of the laboratory). The clinical specimens were analyzed retrospectively (analyzed within the same week after arrival at our laboratory without freezing but stored at 4 °C). A maximum of 12 clinical specimens can be extracted per run. An extraction run needs between 80 and 120 min (depending on the number of clinical specimens, plus placing of materials in the instrument). Blood samples are not recommended for PCR since more blood volume can be applied for blood cultures [14] than for this PCR. Viscous specimens were pretreated according to the protocol.. The Micro-Dx™ system applies UV light at each run for decontamination of the surface of the device to minimize potential contamination associated with the extraction procedure. Additionally, in the PCR setup, UV light was applied at least once a day to minimize potential contamination. Data are available for 803 clinical specimens: 320 biopsies of orthopedic origin (e.g., knee, shoulder, spine, hip, implant, synovia), 175 not further classified biopsies, 118 aspirates (mainly joint), 68 swabs (e.g., wound), 39 sonication (implant), 30 soft tissue (e.g., abscess, cyst), 26 heart-associated biopsies (e.g., aortic valve), 15 liquor, 2 lung biopsies and 10 aspirates pleura. Generally, biopsies of any origin without liquid or buffer are processed based on a previous publication [15] so they can be analyzed by culture (on agar plates as well as enrichment culture) as well as with PCR using the requested 1 mL of available liquid without the need for different procedures for all obtained types of clinical specimens. For prosthesis sonication, a published protocol was used as well [16], enabling parallel culturing and PCR using 1 mL of sonication fluid. Generally, microscopy was not performed for all implant-associated infections based on the specifications of a review [16]. For other clinical specimens, classical Gram staining of original clinical specimens was performed manually with commercial reagents (Biomerieux, Switzerland) [17]. Cultures were identified with MALDI-TOF [18]. Automated culturing is performed with Kiestra TLA (BD, Switzerland) [19–21] using established culture media in clinical microbiology. Specifically, culture of implant-associated infection is also based on published procedures [22, 23].

The only modification of automated DNA isolation was performed by replacing the tube containing β-mercaptoethanol with a tube containing only BugLysis (which is based on the procedure of Sepsitest-UMD [9]) (supplied by Molzym as well) due to potential work safety issue. This replacement limited DNA extraction to bacterial species.

Real time PCR (for 16S rDNA as well as internal control) was performed with PCR reagents supplied with the kit (Molzym) according to their instructions. PCR is SYBR Green-based and includes a melting curve after amplification for the differentiation of specific and nonspecific PCR products. PCR program is identical for internal control as well as 16S rDNA. DNA gel electrophoresis was therefore not needed. According to the package insert, the V3/V4 region was amplified, which was also analyzed in other studies by real time PCR [24]. The PCR product had a length of approximately 450 bp. Two positive controls (supplied by the kit) were used in each run. The internal control DNA (non-16S rDNA) was processed with DNA extraction of clinical specimens but detected as an additional PCR on the same PCR plate. The elution of DNA was 100 μL, and 5 μL thereof was used for PCR on an LC480 (negative control was performed using supplied DNA-free water). The internal control was only relevant for negative clinical specimens, and a Cp > 32 was interpreted as inhibition.

Purification of PCR products was performed with the High Pure PCR Product Purification Kit (order number 11732668001, product of Roche but supplied by Sigma Aldrich) according to the package insert. A maximum of 9.5 μL of purified PCR product was mixed with both sequencing primers (1.25 μL each, supplied by Molzym-kit). The volume of purified PCR product used in Sanger sequencing was dependent on the Cp value. Sanger sequencing (of the total volume of 12 μL) was performed at Microsynth (Balgach, Switzerland). Usually, approximately 400 bp could be analyzed with the obtained chromatogram.

Chromatograms with a single sequence were visually assessed for ambiguities (at the beginning and end of chromatogram), and the sequence was analyzed using “Sepsitest-Blast” with the following link http://www.sepsitest-blast.de/de/index.html. The detailed database of “Sepsitest-Blast” contains more than 7000 quality-controlled reference sequences. Rare species or sequences with homologies < 99% (without wobbles) were additionally analyzed using “NCBI Blast” with the following link: https://blast.ncbi.nlm.nih.gov/Blast.cgi?PROGRAM=blastn&PAGE_TYPE=BlastSearch&LINK_LOC=blasthome, using “nucleotide collection” as a standard database but excluding equivocal entries such as uncultured species.

Mixed chromatograms were analyzed using the online tool Ripseq (Pathogenomix, Santa Cruz, USA with the following link: https://www.ripseq.com/login/login.aspx) [13]. The y cutoff (i.e., signal height of peaks in chromatogram) could be varied, e.g., to exclude small peaks. The tool enables analysis separation of a maximum of 3 different sequences.

The separation of different species was based on sequence diversity. Approximately 1% of the sequence diversity of the 16S rDNA was sufficient to assign a sequence to a specific sequence [25]. Because intragenomic copy variants occur [26], species differentiation should not rely on a variation of only very few bp/400 bp of sequence.

The results were technically (real time PCR and sequencing chromatogram if applicable) and medically (e.g., based on available cultures, patient history, type of clinical specimen) examined by two experienced specialists in the laboratory before reporting to the client as a standard procedure.

It is known that there are groups of closely related species that cannot be distinguished by 16S rDNA sequencing [27, 28]. For PCR-positive, culture-negative samples with many different species with a sequence identity within 1%, only groups (e.g., coagulase-negative staphylococcus, *Streptococcus mitis/oralis* complex) were reported.

## Results

Of 803 clinical specimens, 170 (21.2%) were PCR-positive, and all others were PCR-negative (Table 1). Thirteen (1.6%) clinical specimens were culture-positive and PCR-negative. Twenty-five (3.1%) clinical specimens were positive for enrichment culture, and 595 (74.1%) clinical specimens were negative with both methods.

**Table 1:** Comparison of culture, enrichment culture and PCR for all clinical specimens

	Culture				Total
	positive	only enrichment culture pos.	negative	nd	
**PCR**					
Positive	102	3	62	3	170
Negative	13	25	595	0	633

Out of 803 clinical specimens in total, 170 were PCR-positive, 143 were culture-positive (including enrichment culture), and 595 clinical specimens were negative by culture and PCR

*nd* Not done

**Fig. 1:**
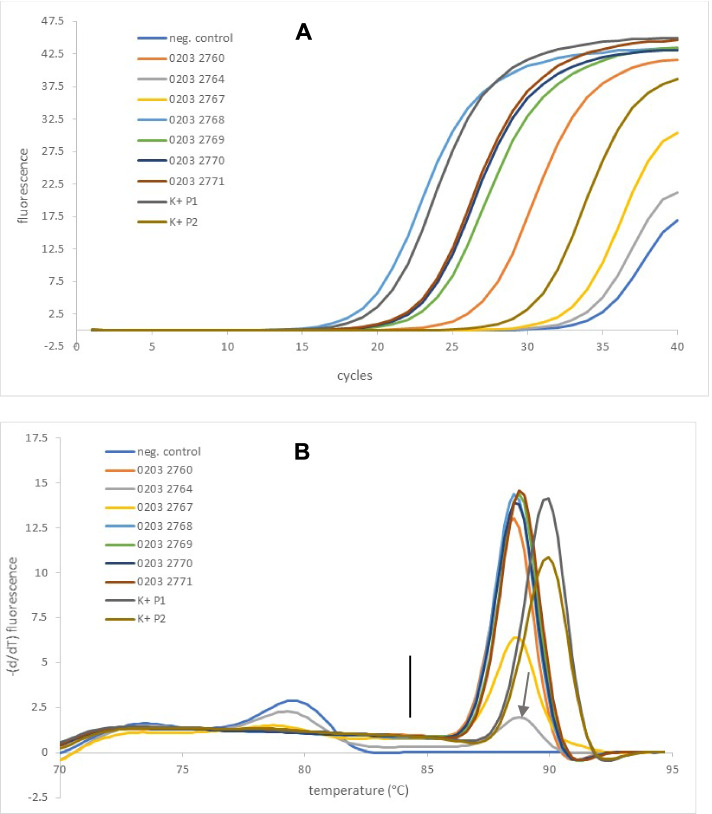
Amplification curves (**A** top) and corresponding melting curve (**B** bottom). Seven PCR-positive clinical specimens from one patient (numbers correspond to anonymized patient identity) as well as 3 controls (P1, P2 as positive control and negative control) are shown. The yellow curve was clearly PCR-positive but negative by culture. The arrow indicates a positive result as well, the result of which from Sanger sequencing was congruent with other clinical specimens of the same patient. The negative control was flat above 84 °C (marked by black line), which indicates the absence of specific PCR products. Peaks below 84 °C indicate nonspecific amplification (e.g., primer dimers). Different Tm values (above 84 °C) are caused by different sequences (in this case, *S. epidermidis* has lower Tm, and *B. subtilis* (present in P1 and P2) has higher Tm

A crucial new point is that PCR-positive results were clearly separable from PCR-negative results in most clinical specimens (Fig. 1). Since there was no strict cutoff regarding the minimal peak height of the melting curve between positive and negative results, sequencing results of those clinical specimens with a low melting peak have to be evaluated very critically (in terms of the technical appearance of the chromatogram derived from Sanger-sequencing, which includes peak height as well as mixed or pure sequence as well as medical interpretation of sequence result) to prevent accidental reporting of an insignificant contamination (which can also occur during sampling). Due to the much faster cycling conditions of real time PCR and its pronounced sensitivity compared to conventional end-point PCR, there is no need for two rounds of PCR (amplification and reamplification) or classical gel electrophoresis, as it was used in the past as an accurate procedure [3]. The turnaround time of the described protocol is very fast. The time to results (from the start of DNA extraction until the end of PCR) was approximately 4 hours for negative specimens. Positive specimens can be reported the next morning of a working day, giving a maximum time to result of 1 day.

Different Tm values indicate different species due to different sequences, and this differentiation has a much lower resolution than Sanger sequencing.

Positive PCR results (*n* = 170) were from 97 different patients (i.e., between 1 and 7 clinical specimens per patient were analyzed). Unfortunately, information on specific treatment with antibiotics is lacking in most cases. It is essential to specify that those clinical specimens that were positive by PCR but negative by culture had large variation of Cp values from the detection limit up to Cp values from 21.56 up to 35.00 (Tables 2 and 3). There were even microscopy-positive specimens that were culture-negative and PCR-positive. This finding is in contrast to clinical specimens with negative PCR but positive culture results. Since DNase also removes DNA from already lysed cells in the original specimen, PCR-positive results are intrinsically from intact cells. The vast majority of PCR-positive specimens had medically relevant results and therefore obviously no contamination. Additionally, the results of a clinical specimen with a positive PCR but negative culture were confirmed by the result of another clinical specimen taken at the same time with a positive culture or a clinical specimen taken previously but also with the same result as PCR.

**Table 2:** Cp values of the 62^a^ truly PCR-positive but culture-negative clinical specimens

**Cp value**	< 22.5	22.5-25.00	25.01-27.5	27.51-30	30.01-32.5	32.51-35.00
**Number**	2	8	6	17	20	8

The diluted positive control (with approx. 100 genome copies/PCR) has a Cp of ~ 31, indicating that only very few (8, i.e., 13%) clinical specimens contain bacterial DNA at the detection limit. The remaining clinical specimens (87%) were abundantly clearly positive. ^a^1 Cp value was retrospectively not available (and is therefore not listed). Negative controls contained no specific amplification product (above 84 °C). For 15 clinical specimens with positive PCR but negative culture information regarding antibiotics were available (13 were treated with an antibiotic, 2 were without an antibiotic). Out of these 13 clinical specimens, 7 had low amount of pathogen DNA (indicated by maximal volume (in μL) of PCR product needed for sequencing)

**Table 3:** Number of observed species determined by PCR

	Number
**Single species**	
*Abiotrophia defectiva*	1
*Arthrobacter aurescens*	1
*Bacillus* sp.	1
*Cutibacterium acnes*^a^	6
*Corynebacterium simulans*	1
*Cardiobacterium hominis*	1
*Clostridium* sp.	1
*Corynebacterium* sp. *(not C. diphtheriae)*	2
*Escherichia coli*	6
*Enterococcus faecalis*	2
*Finegoldia magna*	1
*Fusobacterium necrophorum*	2
*Klebsiella pneumoniae*	1
Coagulase negative Staphylococcus	10
*Haemophilus parainfluenzae*	1
*Mycoabcterium tuberculosis* complex	1
*Moraxella* sp.	1
*Pseudomonas aeruginosa*	1
*Peptoniphilus harei*	2
*Proteus* sp.	3
*Ruminococcus gnavus*	1
*Staphylococcus aureus*	43
*Staphylococcus epidermidis*	37
*Staphylococcus hominis*	1
*Streptococcus dysgalactiae*	14
*Streptococcus agalactiae*	1
*Streptococcus intermedius*	3
*Streptococcus mitis Gruppe*	2
*Streptococcus mutans*	2
*Streptococcus pneumoniae*	1
*Streptococcus pyogenes*	1
*Tetragenococcus halophilus*	1
*Streptococcus pneumoniae/S. pseudopneumoniae*	1
**Mixtures**	
*Anaerococcus* sp.*/Peptoniphilus* sp.	2
*Enterobacter* sp.*/E. coli*	1
*Morganella morganii/Serratia marcescens*	1
*Parvimonas micra/Peptoniphilus* sp.	2
*S. aureus/E. cloacae-Komplex*	1
*S. aureus/P. harei*	6
*S. dysgalactiae/B. fragilis*	2
*S. milleri-group/Fusobacterium periodonticum*	1
*S. milleri-group/Prevotella denticola*	1

Coagulase-negative Staphylococcus was reported only if the culture was negative. In the other cases, the PCR result was adjusted to the culture result identified with MALDI-TOF. Only genus name (e.g., *Enterobacter* sp.) was reported since the sequence could not be assigned to one specific species

^a^Formerly known as *Propionibacterium acnes*

The 25 PCR-negative but enrichment culture-positive specimens contained mainly *Cutibacterium acnes*, *Staphylococcus epidermidis* or *S. aureus* (in 17 clinical specimens). This finding is similar for the 13 clinical specimens with positive culture but negative PCR (8 of 13 were *C. acnes*, *S. epidermidis* or *S. aureus*). The other 12 clinical specimens contained *Proteus mirabilis* (1), *Gemella hemolysans* (1), *Streptococcus mitis/oralis* (1), *S. saccharolytices* (2), *C. avidum* (3), and *Pseudomonas aeruginosa* (5). The 13 culture-positive but PCR-negative samples contained cell numbers of the lowest category [29] in 12 cases (e.g., < 50 cells/mL for sonication), indicating a small difference in the sensitivity of culture and PCR. The results with such a low number of grown colonies have to be interpreted to fit the clinical symptoms.

**Fig. 2:**
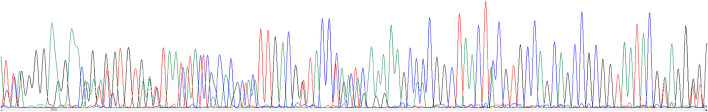
Example of a mixed chromatogram. RiSeq results show the presence of *Citrobacter koseri* and coagulase-negative Staphylococcus (Case 2,111,805,855). Culture confirmed only *Citrobacter koseri*. The material was a biopsy of the malleolus, and the patient was treated with augmentin. Because Sanger sequencing mainly detects concentration differences of approximately less than 1:10 [13], this must be a relevant mixed infection due to a Cp of 29.9 of the amplification. In addition, the same patient had a second clinical specimen with the same mixed sequence. Chromatogram shows stretches of sequence diversity and sequence identity of the two strains. Example is after the 15 months study period

In this study, we observed 42 different species detected by PCR (Table 3). In 17 clinical specimens with positive PCR results, a mixture of two different species was observed. An example of a clearly evaluable and truly positive mixed sequence is shown in Fig. 2. Ripseq indicates if too many sequences are present (prevails mainly for specimens with a result at the detection limit or for unsuitable material with a polymicrobial infection).

There was no evidence for a new species observed among the analyzed clinical specimens based on similarity to reference sequences available in the applied database.

Inhibition was observed for approximately 0.3% of all specimens where extraction failed or PCR was inhibited, and there was not enough volume to repeat the analysis (or send it to another laboratory).

The Cp values and copy number of 16S rDNA per genome were determined according to rrnDB [30]. Approximately 100 genome copies/PCR of *B. subtilis* of a positive control (with 10 copies 16S rDNA per genome) gives a Cp ~ 31. For *A. defectiva*, no information is listed in rrnDB, but the reference strain FDAARGOS_785 contains four 16S rDNA copies. Assuming an amplification efficiency of 2 and including the copy number of 16S rDNA per genome, the highest Cp of 30.44 corresponds to approximately 300 genome copies/PCR, and the lowest Cp of 22.36 corresponds to approximately 250,000 genome copies/PCR. Since 9 of 10 clinical specimens have Cp values lower than 30, the genome copy number corresponds to at least 1000 genome copies/PCR. The analytical sensitivity of detection of different pathogens may be dependent on the copy number of the 16S rDNA per genome [30].

Blood was not tested by PCR since blood cultures are regarded as the “gold standard” [31] and PCR is shown to be of rather low sensitivity [24] compared to culture.

## Discussion

Generally, the analytical sensitivity of culture (enrichment culture excluded) and PCR as well as the specificity of culture and PCR are highly congruent. Specificity is defined as conformity with the cultural identity of the detected pathogen.

Since several specimens per patient were analyzed, discrepant results (e.g., PCR positive but culture negative) can be resolved without any doubt if the same pathogen is present as in other clinical specimens or if there is a previous clinical specimen with a positive culture of the same pathogen. Before reporting results to the clients, available clinical data are taken into account as well. Additionally, real time PCR supports the interpretation of the results due to the Cp values and melting curves. Melting curves support the rapid differentiation of specific and nonspecific PCR products. However, quantitative determination of cells/mL specimens (e.g., for liquor or aspirate), based on Cp values of real time PCR, is not feasible because different organisms contain different 16S rDNA copy numbers [32]. Additionally, the lysis efficiency of different species was not determined.

Since DNase removes most human DNA, bacterial DNA is enriched. It is therefore thought that excess human DNA does not block the binding capacity of the extraction reagents. Ripseq enables the interpretation of mixed sequences, but with a maximum of 3 different species. As an example, slight contamination (which can occur during sampling) can therefore be separated from a clinically relevant strain present with a low copy number.

Of great interest are the cases with suspected endocarditis (Table 4). The detected species were congruent with the literature [33]. Although the specified antibiotics might explain the negative cultures, the detected number of bacteria (based on Cp values and positive microscopy) in the specimen remained high and were therefore not contaminants. Additionally, all three available blood cultures confirm positive PCR. The influence of the duration of treatment with antibiotics and the result of valve cultures (positive or negative) remains complex [33]. Modified Duke criteria (which do not include 16S rDNA PCR) are still widely used for the diagnosis of definite endocarditis [33]. There is evidence that blood culture is incorrectly negative for endocarditis [34], however, with a broad range (of 2 to 71%). From this study, it is assumed that the bacterial cells remained intact despite given antibiotics based on the technology used for DNA extraction [35]. This is supported by the few results of positive microscopy with a negative culture. A recent study [36] declared that antibiotic administration does not influence the recovery of pathogen DNA. Previous literature has confirmed the additional benefit of PCR of infective endocarditis heart valves [37]. However, the mentioned study did not emphasize the use of DNA-free reagents. In any case, our data confirm the need for reliable 16S rDNA PCR supplementary to the use of cultures.

**Table 4:** Results from 10 PCR-positive clinical specimens with suspected endocarditis

Material	Clinical information	PCR	Culture	Antibiotics^b^	Microscopy	Cp and copy number
Valve replacement with vegetations	acute “prosthetic valve endocarditis” after dentist	*Cutibacterium acnes*	negative (blood culture not available)	Augmentin, rimactan, zyvoxid	negative	Cp 30.44 3 copies 16S rDNA
Aortic valve	severe aortic regurgitation with thrombus situation after embolization duration of illness: 4 months	*Cardiobacterium hominis*	negative (blood culture not available)	none	negative	Cp 24.79 3 copies 16SrDNA
Aortic valve	endocarditis with *S.mutans*	*Streptococcus mutans*	negative (blood cultures with *S.mutans* 18 days ago)	Penicillin G, gentamycin	negative	Cp 22.94 5 copies 16S rDNA
Aortic valve	both valves involved mitral valve: PCR negative	*Staphylococus coagulase negative*	negative (blood culture not available)	Floxapen (since 2 days)	negative	Cp 26.70 ~ 6 copies 16S rDNA
Aortic valve	endocarditis aortic and mitral valve	*Staphylococcus epidermidis*	*S. epidermidis* (only in enrichment culture) (blood cultures with *S.epidermidis* 2 and 3 days ago)	Vanco, tazobac	not done	Cp 24.57 6 copies 16S rDNA
Mitral valve	endocarditis mitral valve with *E.faecalis*	*Enterococcus faecalis*	*E. faecalis* (only in anaerobic culture) (blood culture with *E.faecalis* analyzed previously by another laboratory)	Clamoxyl/vancocin	negative	Cp 25.47 4 copies 16S rDNA
Mitral valve^a^	endocarditis mitral valve	*Streptococcus mitis group*	negative (blood culture not available)	no information	gram pos. Cocci ++	Cp 22.36 4 copies 16S rDNA
Tricuspid valve^a^	endocarditis mitral valve	*Streptococcus mitis group*	negative (blood culture not available)	no information	gram pos. Cocci ++	Cp 23.13 4 copies 16S rDNA
Aortic valve	bicuspid aortic valve endocarditis	*Abiotropha defectiva*	negative (blood culture not available)	no information	negative	Cp 27.63 4 copies 16S rDNA
Mitral valve	severe insufficiency of mitral valve	*Streptococcus mutans*	negative (blood culture not available)	no information	gram pos. Cocci ++	Cp 27.35 5 copies 16S rDNA

Clinical information according to the client. Culture includes aerobic incubation on plates, anaerobic incubation on plates and enrichment culture with liquid medium. Previously taken blood cultures are listed additionally. The date of positive blood culture refers to the date of registration of clinical specimens in our laboratory

^a^Same patient

^b^Prior to sending to the laboratory, according to the client

Since information regarding antibiotic treatment are frequently lacking (especially in those specimens other than endocarditis cases), no clear explanation can be given regarding effect of antibiotics for PCR positive but culture negative specimens. Using optimized culture media, sensitivity of culture can be improved despite patient was treated with an antibiotic [22]. This makes comparison of those clinical specimens more complex. In either case, the client obtains a reliable result for a specific treatment.

The terms “false-positive”, “false negative” and the conventional calculation of the specificity are terms that should be critically discussed for this type of PCR. False-positive PCR results are only those due to reagent contamination or contamination during sampling or handling. Significant reagent contamination indicated by the presence of different species in several analyzed clinical specimens (up to 7 in this study) obtained at the same time from the same patient as well as those clinical specimens obtained during the patient’s history was not observed. Additionally, truly positive PCR results of such series were congruent, which shows the benefit of the described procedure (DNA extraction and subsequent PCR). “Analytical sensitivity” and “additional benefit” when using DNA-free reagents for extraction and PCR [24] seem to be more adequate for a broad range of 16S rDNA PCR analyses. The additional benefit is reflected in that 36.5% of the PCR-positive results were negative by culture (Table 1), showing a medically reasonable result, which is supported by other publications [24, 38]. Particularly, this additional benefit can be shown for cases with suspected endocarditis. The culturing procedure used is well established and is obviously not the evident cause for discrepant results.

Generally, there are different reasons for culture-negative but PCR-positive results [24].

For orthopedic specimens analyzed in this study, *S. aureus*, *S. epidermidis* and coagulase-negative *Staphylococcus* were the most abundant species, followed by *C. acnes,* which is congruent with previous findings [38] and *S. dysgalactiae*. Although *S. dysgalactiae* has been increasingly recognized as an important human pathogen [39], it seems to be rare in orthopedic specimens [40]. For those clinical specimen with positive enrichment culture but negative PCR it is strongly assumed that this is due to the fact that enrichment culture is more sensitive than standard culture in the context of orthopedic clinical specimens with e.g. *Cutibacterium acnes*, *S.epidermidis* and *S.aureus* [41, 42]. Even for group B streptococcus, enrichment culture of rectovaginal swabs is the primary means to detect colonization [43]. However, enrichment culture requires up to 14 days for growth [42]. This is much slower than the evaluated molecular method. For an optimized diagnostic procedure, the use of the evaluated molecular method and the two types of cultures gives a high sensitivity and an appropriate turnaround time.

Exact quantification determination of the whole procedure (DNA extraction and real time PCR) was not regarded as reasonable. Therefore, experiments for testing extraction efficiency using artificially spiked specimens were not performed, since this does not exactly match real clinical specimens (which include biofilms and use of antibiotics). However, semiquantitative information of real time PCR is described without the need for specific statistical methods.

Obviously, costs for consumables are higher than other automated extraction methods e.g., [44]. The preferred method remains the decision of the user. Nevertheless, the described method clearly shows that it is fast and provides unequivocal results (for the vast majority of clinical specimens) and is therefore a pronounced improvement over past methods and regarded as adequate for use in molecular routine diagnostics of clinical specimens from sterile body sites. Sequencing results from very small peaks of the melting curve (Fig. 1, Panel B) still have to be examined critically in technical terms and in combination with clinical information and parameters of infection.

## Data Availability

Sequencing results were compared to public databases (NCBI Blast, Sepsitest-Blast or Ripseq), and no new sequences of undiscovered species were found. All results were mentioned in the text and links to used databases are present in the manuscript.
